# A(H1N1)pdm09 influenza infection: vaccine inefficiency

**DOI:** 10.18632/oncotarget.16459

**Published:** 2017-03-22

**Authors:** Nehemya Friedman, Yaron Drori, Rakefet Pando, Aharona Glatman-Freedman, Hanna Sefty, Ravit Bassal, Yaniv Stein, Tamy Shohat, Ella Mendelson, Musa Hindiyeh, Michal Mandelboim

**Affiliations:** ^1^ Central Virology Laboratory, Ministry of Health, Chaim Sheba Medical Center, Ramat-Gan, Israel; ^2^ Department of Epidemiology and Preventive Medicine, School of Public Health, Sackler Faculty of Medicine, Tel-Aviv University, Tel-Aviv, Israel; ^3^ The Israel Center for Disease Control, Israel Ministry of Health, Tel-Hashomer, Israel; ^4^ Departments of Pediatrics and Family and Community Medicine, Valhalla, New York, USA

**Keywords:** influenza A, H1N1, vaccine, Clade 6B

## Abstract

The last influenza pandemic, caused by the swine A(H1N1)pdm09 influenza virus, began in North America at 2009. Since then, the World Health Organization (WHO) recommended integration of the swine-based virus A/California/07/2009 strain in yearly vaccinations. Yet, infections with A(H1N1)pdm09 have continued in subsequent years. The reasons for this are currently unknown. During the 2015–2016 influenza season, we noted an increased prevalence of A(H1N1)pdm09 influenza virus infection in Israel. Our phylogenetic analysis indicated that the circulating A(H1N1)pdm09 strains belonged to 6B.1 and 6B.2 clades and differed from the vaccinating strain, with approximately 18 amino acid differences found between the circulating strains and the immunizing A/California/07/2009 strain. Hemmaglutination inhibition (HI) assays demonstrated higher antibodies titer against the A/California/07/2009 vaccinating strain as compared to the circulating Israeli strains. We thus suggest that the current vaccination was not sufficiently effective and propose inclusion of the current circulating A(H1N1)pdm09 influenza viruses in the annual vaccine composition.

## INTRODUCTION

Influenza viruses are responsible for many respiratory infections affecting all age groups, particularly the elderly population [1, 2]. Annual epidemics of seasonal influenza viruses are estimated to result in approximately three to five million cases of severe illness and about 250,000 to 500,000 deaths worldwide (WHO, 2014). In addition, influenza pandemics, that occur every few decades, pose a major global threat.

Influenza virus genome contains 8 single-stranded RNA molecules, which encodes at least 13 proteins, among them Neuraminidase (NA) and Hemagglutinin (HA), which are expressed on the surface of infected cells [3–5]. To avoid immune recognition, both the HA and the NA proteins are subjected to extensive antigenic changes, complicating the design of a universal vaccine aiming to target all influenza strains [6]. Thus, the primary preventive strategy currently available against influenza virus infection is a vaccine that is designed and administered each year [7]. The vaccine is composed of three or four influenza virus strains prevailing in the influenza season of the opposite hemisphere.

The most recent influenza pandemic occurred in 2009. Although the mortality rate was relatively low, it affected many people around the world [8]. The pandemic began in La Gloria, Mexico in February 2009 [9]. Since that time, the A(H1N1)pdm09 virus spread rapidly across the globe and early assessments of transmissibility and severity were made [9]. In 2010, the WHO announced that A(H1N1)pdm09 virus is expected to continue circulating as a seasonal virus [10]. Indeed, infections with the virus were noted in subsequent years, accounting for 24.5% of the 2010–2014 infections in North China, 16% of which were in 2013–2014 [11]. In 2012–2013, 28.5% of the influenza-infected individuals in France, Italy, Spain and Lithuania together, were infected with influenza A(H1N1)pdm09 [12]. In Austria, 18.3% of the influenza-infected individuals were A(H1N1)pdm09-positive in winter 2014–2015 [13]. During the post-pandemic period in Israel, 10% of the patients hospitalized in 2010–2011 winter season and 5% of those hospitalized in the 2011–2012 winter season, were infected with the A(H1N1)pdm09 virus [6]. These numbers rose in winter 2012–2013, when 20% of the hospitalized patients were infected with the A(H1N1)pdm09 influenza virus, 64.8% of whom were pregnant women [6]. A recent Eurosurveillance report estimated that the northern hemisphere vaccine still offers protection against circulating A(H1N1)pdm09 viruses, because only limited drift has been observed [14].

Here, we report of a significant A(H1N1)pdm09 Influenza virus infection rate in the last winter season (2015–2016) in Israel, analyze the virus properties and demonstrate that it differs from the strain included in the vaccine.

## RESULTS

### A(H1N1)pdm09 prevalence in Israel during the 2015–2016 winter season

**Figure 1:**
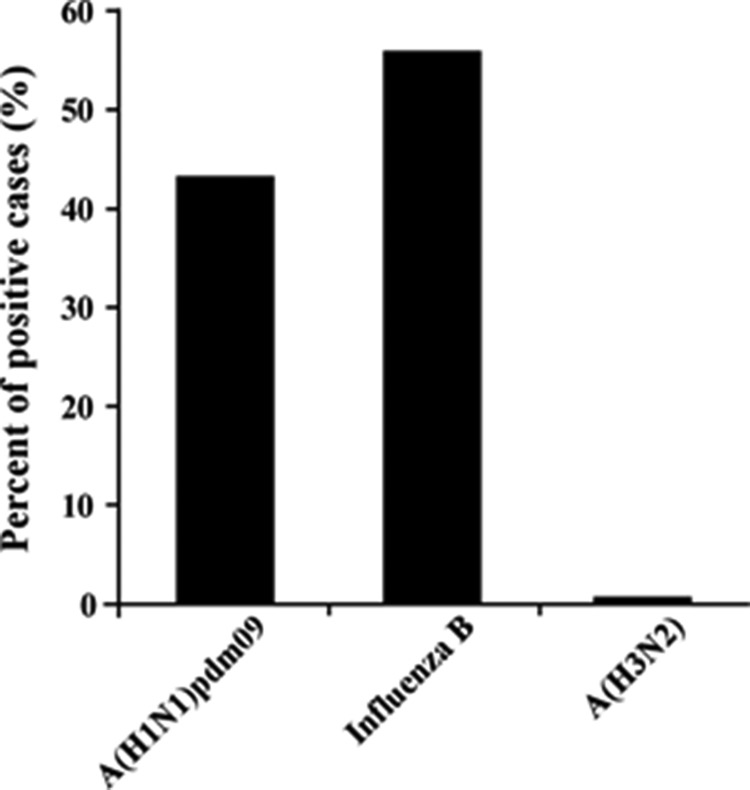
Percentages of influenza viruses Distribution of influenza viruses strains (X axis) among 1917 influenza-positive cases in 2015–2016 winter season.

Samples of patients with Influenza-like illness (ILI) were obtained from 26 clinics around Israel, between September 2015 until March 2016. Among the tested samples, 45.12% (865 patients) proved positive for the presence of influenza viruses. Of these, 56.3% were positive for influenza B, 43.01% for A(H1N1)pdm09, and only 0.7% for influenza A(H3N2) (Figure 1). Interestingly, of the A(H1N1)pdm09-infected individuals (372 patients), 14.5% (54 patients) had received the influenza vaccine. More intriguingly, among the A(H1N1)pdm09 negative ILI patients (1545 patients), only 12.5% (194 patients) were vaccinated. Upon analysis of the weekly distribution of A(H1N1)pdm09 infection in 2015–2016, we noted that the infection began in week 48 of 2015, peaked in the 3rd week of January 2016 and persisted until the end of March (week 12, 2016, Figure 2). When compared to influenza seasons 2013–2014 and 2014–2015, the 2015–2016 winter season was associated with a significant increase in A(H1N1)pdm09 infection-related infection (Figure 3).

**Figure 2:**
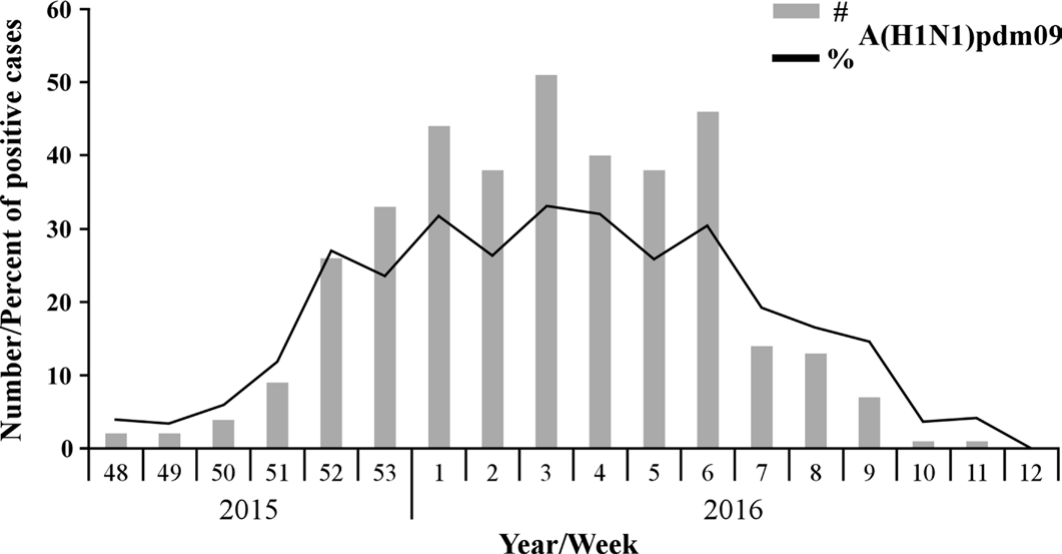
Distribution of A(H1N1)pdm09 infection in the 2015–2016 winter season Number (#) and percent (%) of A(H1N1)pdm09-positive cases among 1917 samples collected during the 2015–2016 winter influenza season, starting from the 48th week of 2015 until the 12th week of 2016.

**Figure 3:**
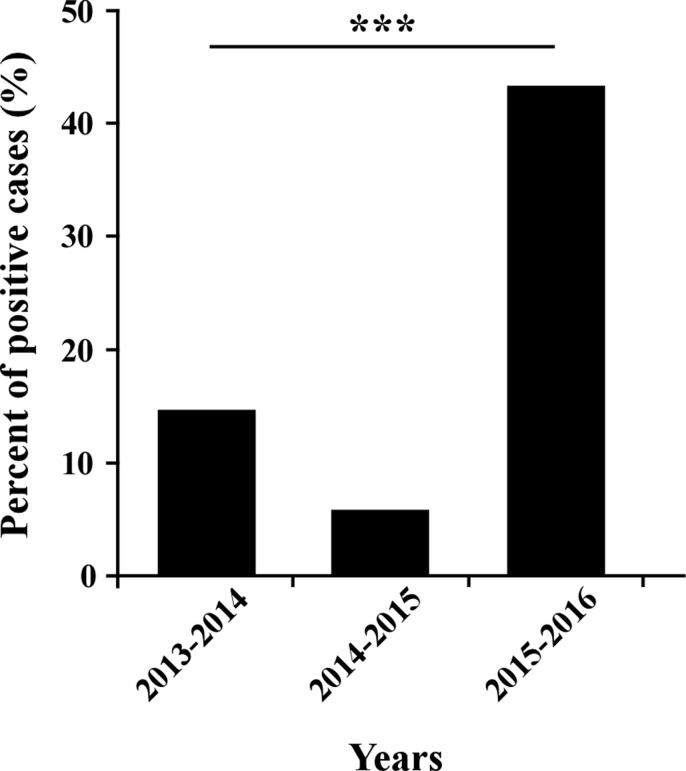
Percent of A(H1N1)pdm09 infection over the years The annual percent of patients infected with the A(H1N1)pdm09 virus in winter seasons 2013–2014 (14.71% of 503 positives for Influenza), 2014–2015 (5.81% of 327) and 2016 (43.01% of 865). *** indicates *P* < 0.0001 using the chi-square test.

### Genetic variations of seasonal A(H1N1)pdm09 viruses

**Figure 4:**
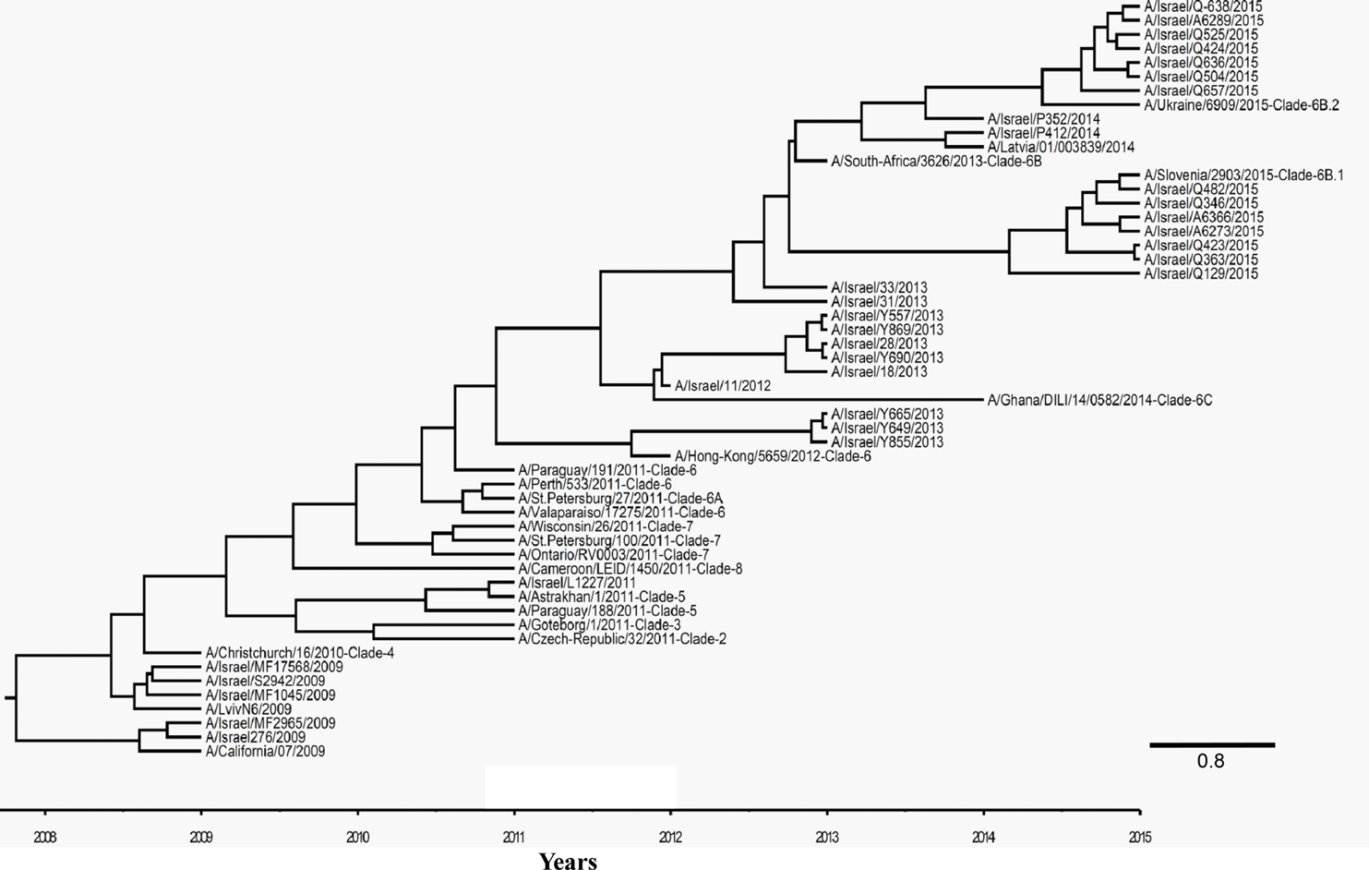
Phylogenetic tree of A(H1N1)pdm09 viruses Bayesian maximum-clade-credibility time-scaled phylogenetic tree (BEAST), generated using 54 A(H1N1)pdm09 Influenza HA gene, obtained from reference genes and patient samples collected between 2009 and 2016 in Israel. Alignment was observed for 1035 base pairs of all the genes in the phylogenetic tree. Clade number is indicated next to the reference viruses. Scale bar display time in years.

Several A(H1N1)pdm09 viruses were randomly selected from 2015–2016 winter period samples and from samples collected during previous winter seasons, to enable a whole *HA* gene sequencing (1701 bp, 566 amino acid) and construction of a phylogenetic tree. Some of those viruses were isolated from individuals vaccinated against the California/07/2009 strain. We aligned 1035 base pairs of all the sequences in the phylogenetic tree. The resulting cladogram indicated that while the vaccinating strain A/California/07/2009 belongs to clade 1, the circulating Israeli strains belonged to clade 6B, represented by A/South-Africa/3623/2013 (Figure 4). The Israeli strains belong either to clade 6B.1 or 6B.2, represented by A/Slovenia/2903/2015 or A/Ukraine/6909/2015 strains, respectively (Figure 4).

**Figure 5:**
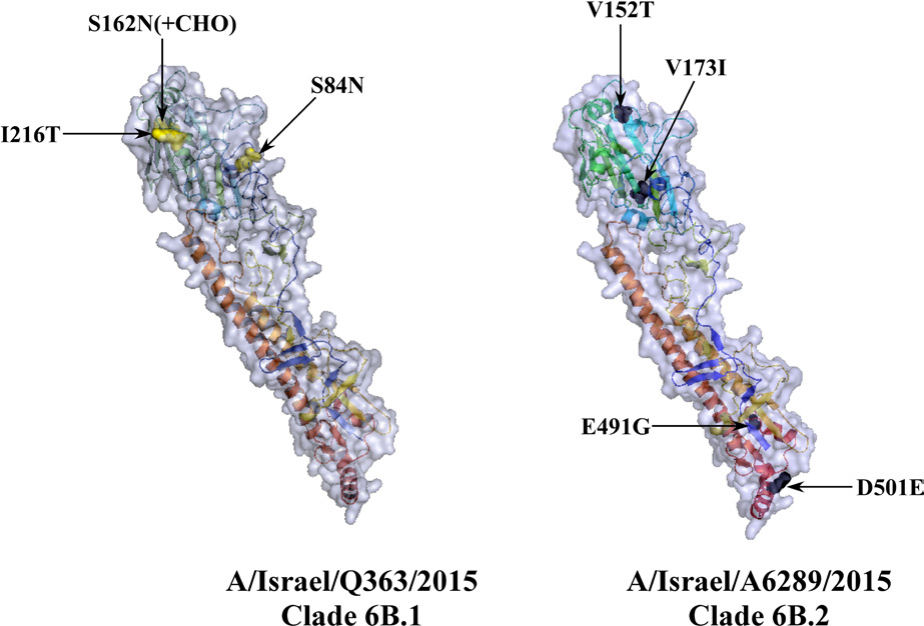
Three dimensional presentation of HA protein of circulating 6B clade Israeli viruses *HA* gene sequences of A/Israel/Q363/2015 (clade 6B.1) and A/Israel/A6289/2015 (clade B.2) translated using ExPASy translate tool and their 3D structure predicted using SWISS-MODLE server. The tertiary structure designed in PyMOL. Mutations which define 6B.1 (yellow) or 6B.2 clade (black) are marked in arrow.

Comparison of the *Hemagglutinin* sequences of the vaccine strain to those of the circulating Israeli strains belonging either to clade 6B.1 or to 6B.2 (represented by A/Israel/Q363/2015 and A/Israel/A6289/2015, respectively), identified 16 and 18 differences in the amino acid sequences, respectively (Table 1). All 6B clade viruses in our analysis contained the amino acid variations that define this clade: D97N, K163Q, S185T, S203T, A256T and K283E (Table 1) [15–17]. Additional mutations included S84N, S162N and I216T in the 6B.1 clade and V152T, V173I, E491G, D501E in the 6B.2 clade [18]. The last 7 mutations are located on the Hemagglutinin surface (Figure 5). There are four major antigenic sites on the Hemagglutinin protein named -Sa, Sb, Ca, and Cb [19, 20]. Mutations K163Q, S185T, S203T (clade 6B) are respectively located on Sa, Sb and Ca1 antigenic sites. In addition, we found that S162N (clade 6B.1) located in Sa site [21] and V173I (clade 6B.2) in Ca1 site [15].

**Table 1:** Mutations sequenced in Israeli 6B clade viruses

Amino Acid position	A/California/07/2009 vaccine		A/Israel/Q363/2015 Clade 6B.1		A/Israel/A6289/2015 Clade 6B.2	
83	CCT	P	TCT	S	TCT	S
84	AGT	S	AAT	N	AGT	S
97	GAT	D	AAT	N	AAT	N
152	GTT	V	GTT	V	ACT	T
162	AGC	S	AAC	N	AGC	S
163	AAA	K	CAA	Q	CAA	Q
173	GTC	V	GTC	V	ATC	I
185	AGT	S	ACT	T	ACT	T
187	GAG	E	GAC	D	GAC	D
203	TCA	S	ACA	T	ACA	T
216	GCA	A	ACA	T	ATA	I
224	GCA	A	GAA	E	GAA	E
256	GCA	A	ACA	T	ACA	T
273	CAC	H	CAC	H	CAC	H
283	AAG	K	GAG	E	GAG	E
321	ATC	I	GTC	V	GTC	V
374	GAG	E	AAG	K	AAA	K
451	AGC	S	AAC	N	AAC	N
491	GAG	E	GAG	E	GGG	G
499	GAA	E	AAA	K	AAA	K
501	GAT	D	GAT	D	GAA	E
Total Differences				**16**		**18**

A list of mutations and their amino acid position as found in sequencing 6B.1 (A/Israel/Q363/2015) and 6B.2 (A/Israel/A6289/2015) clade viruses, in comparison to the vaccine strain (A/California/07/2009).

### Immunity to old and new A(H1N1)pdm09 antigens

**Figure 6:**
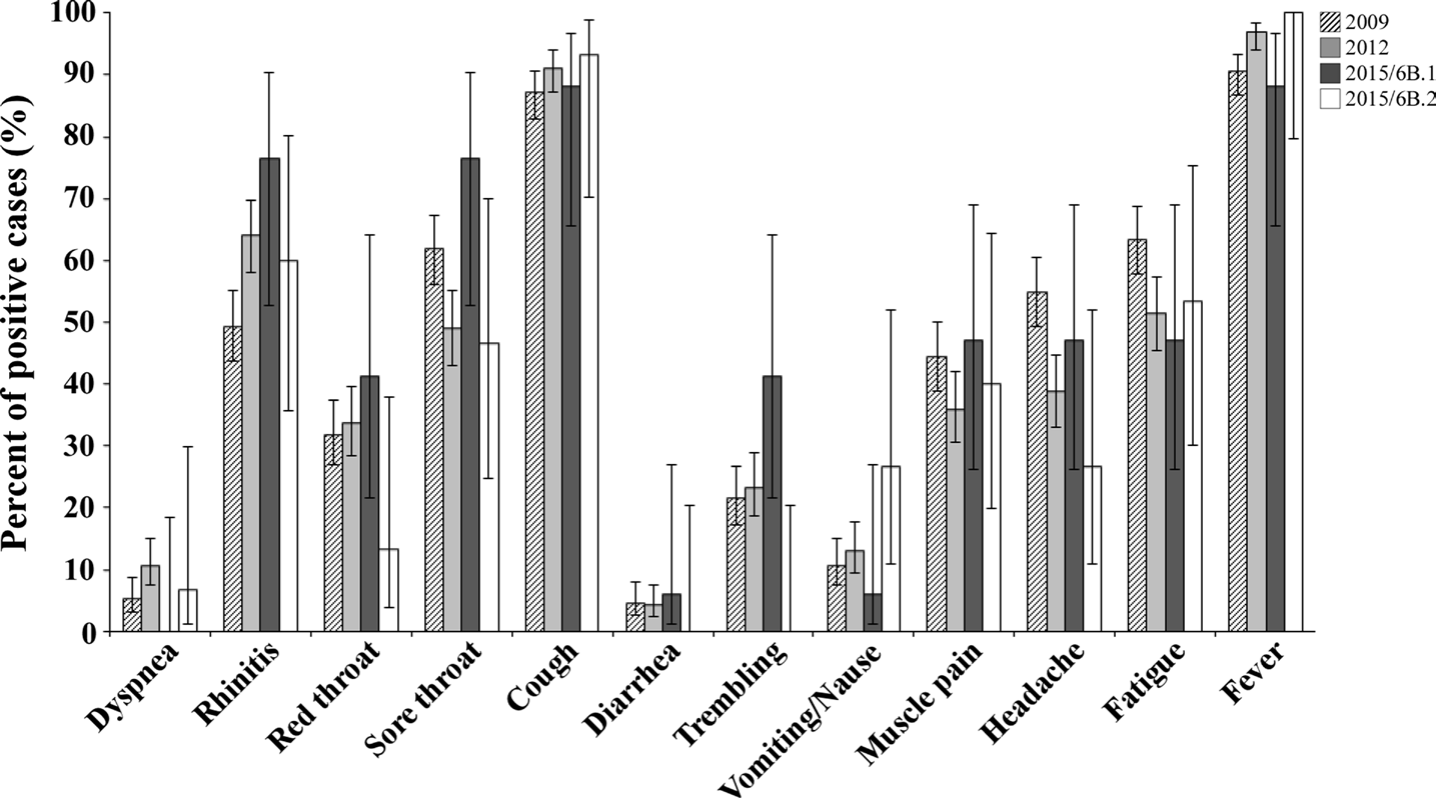
Clinical characteristics of patients infected with 6B clade A(H1N1)pdm09 viruses A summary of the clinical symptoms of Israeli patients infected in 2015–2016with A(H1N1)pdm09 virus belonging to the 6B.1 or 6B.2 clade.

**Figure 7:**
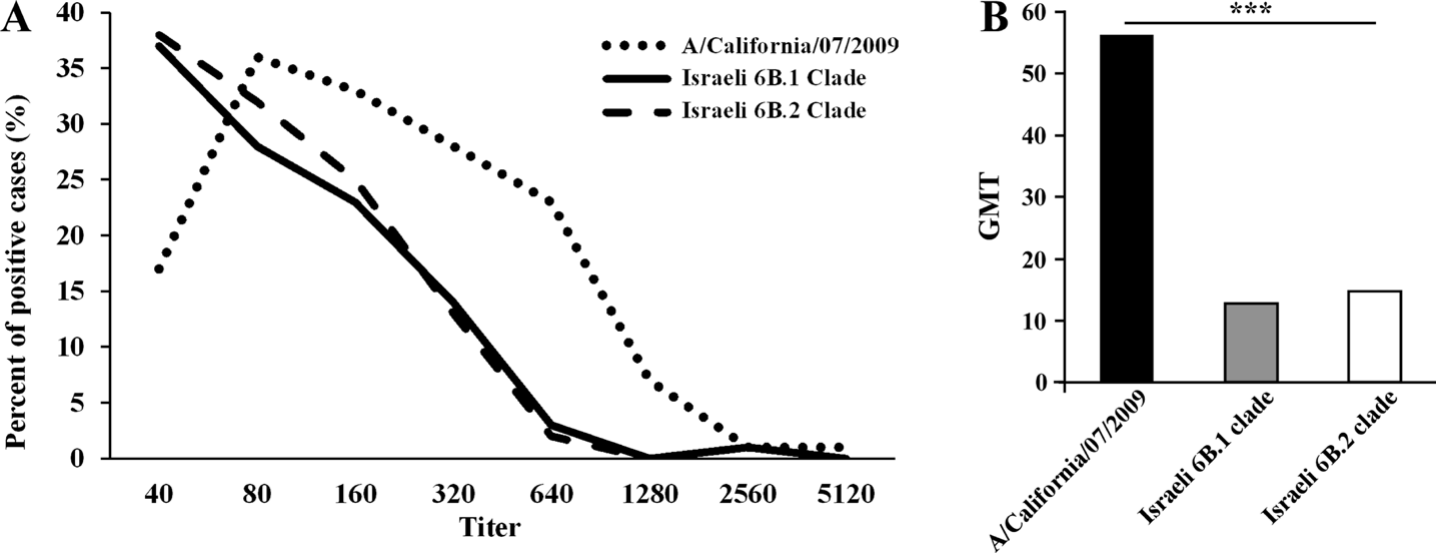
Antibodies against 6B clade antigens were not dominant in 2014 The presence of antibodies against A/California/07/2009, Israeli 6B.1 and 6B.2 antigens was detected using the HI test. The figure shows the distribution of the positive cases by antibody titer (**A**) and their geometric mean (**B**). *** indicates *P* < 0.0001 using the Kruskal-Wallis test.

Analysis of the symptoms reported by patients infected with A(H1N1)pdm09 virus in 2009 and 2012 versus 2015th circulating 6B clade strains, yielded no significant differences between the patient groups (Figure 6). In addition, to evaluate the potential of serum antibodies collected from individuals to cross-react with the influenza A(H1N1)pdm09 circulating strains, sera from 240 individuals obtained in 2014–2015, prior to the appearance of the current strains. Sera samples were applied in HI assays against the vaccine strain A/California/07/2009 antigen and against two circulating influenza A(H1N1)pdm09 antigens from clade 6B.1 and 6B.2. As seen in Figure 7, the vaccine strain was better recognized (Figure 7A), and yielded a higher geometric mean of antibodies titer (GMT Figure 7B), as compared with the circulating Israel strains.

## DISCUSSION

The recent years gave us the opportunity to study the evolution and spread of the pandemic A(H1N1)pdm09 Influenza virus, since the time it first appeared in 2009 until today. At the post-pandemic period, until 2015, HA genes have evolved and eight genetic groups have been designated [6]. Since September 2015, most A(H1N1)pdm09 viruses fell into genetic group 6B and in two emerging subgroups, 6B.1 and 6B.2 [18]. Despite the emergence of new genetic subgroups, a report for the WHO suggested in February 2016 that these 6B clade viruses are antigenically similar to the vaccine virus A/California/7/2009 [18].

During the 2015–2016 influenza season at total of 1548 outpatient samples were negative for influenza, of which 194 (12.5%) of them were vaccinated against influenza. To test the vaccination efficiency we compared the odds ratio of vaccinated and non-vaccinated individuals among individuals who were positive and negative for influenza. The odds ratio was 1.18, indicating no difference between the vaccinated and the none-vaccinated individuals. The circulating Israeli strains were shown phylogenetically differ from the vaccinating A/California/7/2009 A(H1N1)pdm09 strain, with the vaccinating strain belonging to clade 1, and the circulating strains belonging to clades 6B.1 and 6B.2. Furthermore, approximately 18 amino acid modifications were identified between the circulating Israeli strains and the vaccinating strain. Some of these mutations are located in antigenic sites [15, 19–21], and therefore might result in ineffective immunogenic recognition.

Laboratories in the United States and United Kingdom have recently reported maintained effectiveness of the influenza vaccine bearing the A/California/7/2009 strain [22, 23]. However, in contrast to these reports, which used post-infected ferret's sera in their HI assays, we directly compared the HI potential of A/California/7/2009 versus Israeli 6B viruses isolated from patient samples, to identify cross-protecting antibodies in the Israeli population. This analysis significantly demonstrate that the anti-influenza antibodies recognize the circulating Israeli 6B strains 4-fold less efficiently as compared to the vaccinating strain. In line with these findings, the WHO report including recommendations for the northern hemisphere 2015–2016 vaccine composition, states that the GMTs in HI against representative 6B clade viruses were significantly reduced compared to HI titers to the vaccine virus, particularly in pediatric serum panels [18].

In the past seven years, the A/California/7/2009 strain has been included in the influenza vaccine [24]. However, we show here that the circulating A(H1N1)pdm09 viruses substantially differ from the vaccinating strains and are not adequately recognized by the vaccine strains. In view of these results, we suggest that current circulating strains should be included in the vaccine.

## MATERIALS AND METHODS

### Patients and samples

As part of community influenza surveillance, conducted in collaboration with the Israel Center for Disease Control (ICDC), respiratory clinical samples (nose-throat swabs) were collected from 1917 patients presenting with Influenza-like illness (ILI), during the winter season spanning between October 2015 and April 2016.

Serum samples (*N* = 240) were obtained from the Israel National Serum Bank established by the Israel Center for Disease Control. The samples were collected from healthy individuals in 2014.

### Detection of A(H1N1)pdm09 virus

Viral genomic RNA was extracted from patient samples using the NucliSENS easyMAG (BioMerieux, France). Influenza A, influenza B and A(H1N1)pdm09 strains were identified using a panel of real-time reverse transcription PCR (rRT-PCR), as previously described [25, 26].

### Sample preparation for sequencing

For the phylogenetic analysis, influenza *Hemagglutinin* gene-specific primers (WHO Swine genome set [27] were amplified using the OneStep RT-PCR Kit (QIAGEN). ExoProStar (Illustrsa) was activated on the cDNA to clean unincorporated primers and dNTPs. The Big Dye^®^ Terminator v1.1 cycle kit (ABI Prism^®^) was used for sample sequencing, as per the manufacturer's instructions. The BigDye^®^ Xterminator Purification Kit (ABI) was then used to clean the cDNA. Samples were processed and sequenced in a Genetic Analyzer 3500 (ABI\HITACHI) machine.

### Phylogenetic analysis

For the phylogenetic analysis, Influenza *Hemagglutinin* gene-specific primers were used [6, 27]. The Sequencher^®^ 5.0 program (Gencodes Corporation, Ann Arbor, MI) was used to compare the nucleotide sequences of the collected samples to *HA* genes of various Influenza A clades.

The evolutionary relationships and the most recent common ancestor (MRCA) for the influenza A(H1N1)pdm09 virus *Hemagglutinin* sequences, were calculated using a Bayesian Markov chain Monte Carlo (MCMC) method, applied using a relaxed molecular clock, as implemented in the BEAST program (version 1.7.5). Trees were visualized and edited with the Figure Tree program (version 1.4.2) included in the BEAST software package [28].

### Hemagglutination inhibition (HI) analysis

All sera were treated (16 h) with receptor destroying enzyme (RDE) (Sigma C8772), diluted 1:4, prior to heat inactivation (30 min, 56°C). Absorption with erythrocytes was then conducted to remove non-specific binding, in accordance with a WHO-recommended protocol [29]. Two-fold serial dilutions (1:40–1:5120) of sera in 25 μl PBS were prepared in V-shaped well plates, and an equal volume of four HA units was added. The mixture was then incubated at room temperature for 1 h. Fifty microliters of 0.5% chicken erythrocytes suspended in PBS, were added to the wells, and mixed by shaking the plates on a mechanical vibrator. Agglutination patterns were read after 30 min and the HI titer was defined as the reciprocal of the last dilution of serum that fully inhibited Hemagglutination. The cut-off value for a positive result was set at 1:40. The influenza A(H1N1)pdm09 (A/California/07/2009 NYMC X-179A) antigen was supplied by the WHO. The Israeli 6B.1 and 6B.2 clades antigens were A/Israel/Q363/2015 and A/Israel/Q504/2015, respectively.

### Statistical analysis

The annual percent of positive cases was calculated by dividing the number of positive A(H1N1)pdm09 samples by the total number of positive tests for Influenza Viruses. The Chi-Square test was applied to evaluate the differences in positivity percent between the compared years. To calculate significant differences of HI results, we used Kruskal-Wallis test (One-way ANOVA on ranks), since the titers have non-normal distributions. All analyses were performed using SPSS. A *p*-value < 0.05 was considered to be statistically significant.

### Ethical considerations

Community influenza surveillance, including nose-throat swabs, is performed under the Public Health Ordinance enacted in Israel and thus informed consent was not required. The IRB of the Sheba Medical Center approved the research (Helsinki Number 2873-15-SMC for molecular characterization and 1967-15-SMC for serologic studies).

This work was performed in partial fulfillment of the requirements for a Ph.D degree of Nehemya Friedman, Sackler Faculty of Medicine, Tel Aviv University, Israel.
